# Real-time Symptom Assessment in Patients With Endometriosis: Psychometric Evaluation of an Electronic Patient-Reported Outcome Measure, Based on the Experience Sampling Method

**DOI:** 10.2196/29480

**Published:** 2023-03-03

**Authors:** Esther van Barneveld, Arianne Lim, Nehalennia van Hanegem, Frits van Osch, Lisa Vork, Joanna Kruimel, Marlies Bongers, Carsten Leue

**Affiliations:** 1 GROW School of Oncology and Developmental Biology, Maastricht University Maastricht Netherlands; 2 Department of Gynaecology and Obstetrics, Maastricht University Medical Center+ Maastricht Netherlands; 3 Department of Reproductive Medicine and Gynecology, University Medical Center Utrecht Utrecht Netherlands; 4 NUTRIM School of Nutrition and Translational Research in Metabolism, Maastricht University Maastricht Netherlands; 5 Department of Clinical Epidemiology, VieCuri Medical Center Venlo Netherlands; 6 Department of Internal Medicine, Division of Gastroenterology and Hepatology, Maastricht University Medical Center+ Maastricht Netherlands; 7 Department of Gynaecology and Obstetrics, Máxima Medical Center Veldhoven Netherlands; 8 School for Mental Health and Neuroscience Maastricht University Maastricht Netherlands; 9 Department of Psychiatry and Psychology, Maastricht University Medical Center+ Maastricht Netherlands

**Keywords:** endometriosis, pelvic pain, positive affect, negative affect, patient-reported outcome measure, momentary symptom assessment, experience sampling method, pain, PROM, outcome, patient-reported, assessment, symptom, sampling, method, evaluation, psychometric, real-time, prospective

## Abstract

**Background:**

The experience sampling method (ESM) holds advantages over traditional retrospective questionnaires including a high ecological validity, no recall bias, the ability to assess fluctuation of symptoms, and the ability to analyze the temporal relationship between variables.

**Objective:**

This study aimed to evaluate the psychometric properties of an endometriosis-specific ESM tool.

**Methods:**

This is a short-term follow-up prospective study, including patients with premenopausal endometriosis aged ≥18 years who reported dysmenorrhea, chronic pelvic pain, or dyspareunia between December 2019 and November 2020. An ESM-based questionnaire was sent out by a smartphone application 10 times a day during 1 week on randomly chosen moments. Additionally, patients completed questionnaires concerning demographics, end-of-day pain scores, and end-of-week symptom scores. The psychometric evaluation included compliance, concurrent validity, and internal consistency.

**Results:**

Twenty-eight patients with endometriosis completed the study. Compliance for answering the ESM questions was as high as 52%. End-of-week pain scores were higher than ESM mean scores and showed peak reporting. ESM scores showed strong concurrent validity when compared with symptoms scored by the Gastrointestinal Symptom Rating Scale–Irritable Bowel Syndrome, 7-item Generalized Anxiety Disorders Scale, 9-question Patient Health Questionnaire, and the majority of questions of the 30-item Endometriosis Health Profile. Cronbach α coefficients demonstrated a good internal consistency for abdominal symptoms, general somatic symptoms, and positive affect, and an excellent internal consistency for negative affect.

**Conclusions:**

This study supports the validity and reliability of a newly developed electronic instrument for the measurement of symptoms in women with endometriosis, based on momentary assessments. This ESM patient-reported outcome measure has the advantage of providing a more detailed view on individual symptom patterns and offers the possibility for patients to have insight in their symptomatology, leading to more individualized treatment strategies that can improve the quality of life of women with endometriosis.

## Introduction

Endometriosis is defined as an estrogen-dependent condition with endometrium-like tissue outside the uterus [1]. The prevalence has been estimated at around 10% in women of reproductive age and up to 50% in women with chronic pelvic pain (CPP) or fertility problems [2,3]. Dysmenorrhea, CPP, dyspareunia, fatigue, and infertility are the leading symptoms [4,5]. Therefore, endometriosis is of significant social and psychological impact, decreasing patients’ quality of life. Furthermore, the annual economic burden of women with endometriosis is high [6].

Endometriosis is currently managed by surgical or medical interventions; however, approximately 50% of women with endometriosis have recurrent symptoms over a period of 5 years, irrespective of the treatment approach [7]. The anatomical stage of endometriosis is not directly related to the degree of symptoms [8], which has led to the suggestion that the perception and recurrence of symptoms may be influenced by other factors, such as psychological distress [9-11]. Therefore, accurate symptom assessment, including patient-specific factors that could influence perceived endometriosis symptoms, is of utmost importance. Currently, there is no tool available that assesses symptoms and concurrently takes into account social and psychological factors, which could be of aid in the process toward a more individualized therapeutic approach. Furthermore, outcome measures for endometriosis symptoms concern retrospective questionnaires like the widely used 30-item Endometriosis Health Profile (EHP-30) [12]. A shorter recall period reduces recall bias, and the use of repeated measurements allows evaluating the variability and the effect of time‐varying factors between multiple measurements on the outcome.

An endometriosis-specific patient-reported outcome measure (PROM) that uses the experience sampling method (ESM) has recently been developed at the Maastricht University Medical Center+ (MUMC+) [13]. This instrument provides a detailed insight into symptom patterns and helps in personalizing treatment strategies and, consequently, can aid in making meaningful changes to women’s lives. The ESM is an electronic momentary assessment method characterized by randomly repeated self-reports in real-time moments that holds several advantages over traditionally used measurement tools, such as a high ecological validity and the ability to exclude recall bias as it is not relying on long-term memory [14]. The ESM offers the possibility to monitor clinically relevant experiences, such as pain, mood, and behavior, in the context of daily life and offers the ability to monitor fluctuation in symptoms to allow the assessment of the temporal relationship between variables [14-16]. It enables the assessment of various constructs and psychological mechanisms, for example, stress sensitivity and coping, which are difficult to assess using cross-sectional questionnaires [17]. It has frequently been used in psychiatric patients, and the evidence regarding the ESM in somatic illnesses is growing [18-24]. This study aimed to evaluate the psychometric properties of the above-mentioned endometriosis-specific ESM tool.

## Methods

### Ethics Approval

This prospective study was approved by the medical ethics committee of MUMC+ (Ref. No. 2019-1261).

### Study Participants

Patients with premenopausal endometriosis aged ≥18 years, diagnosed by physical examination, imaging techniques, or laparoscopy, were recruited at the outpatient gynecology department at MUMC+, a tertiary referral center, between December 2019 and November 2020. Furthermore, patients were recruited by advertisement on the website of the Dutch Endometriosis Foundation. Subjects were eligible for inclusion if they reported one of the endometriosis-related pain symptoms (ie, dysmenorrhea, pelvic pain, or dyspareunia) on average at least 1 day per week during the last 3 months. Pregnant women and patients with any other organic explanation for CPP were not eligible for participation. Participants had to be able to understand written Dutch, and written informed consent was obtained from all participants before participation.

### Data Collection

#### Overview

At baseline, patients were asked to fill in an electronic clinical case report form (eCRF) concerning demographic information and medical history in Castor electronic data capture (EDC) [25]. Then, during the 7-day study period, subjects completed repeated real-time questionnaires using the ESM tool and an end-of-day questionnaire. At the end of the study period, several retrospective questionnaires were completed.

#### ESM Questionnaire

The ESM questionnaire contains items concerning endometriosis symptoms, general somatic symptoms, psychological symptoms, and contextual/social information as well as questions concerning nutrition and medication. Depending on previous answers on trigger questions, this questionnaire comprises a minimum of 31 and a maximum of 42 items. Additionally, there is a morning questionnaire that includes questions concerning sleep and sexual behavior, which comprises a minimum of 4 and a maximum of 7 questions [13]. Most questions are scored on an 11-point Numeric Rating Scale (NRS; 0=*not at all* to 10=*very much so*). However, some questions were scored on a scale from –5 to +5 (0=*neutral*). The development of this ESM questionnaire for patients with endometriosis was previously described [13]. The complete questionnaire was officially translated from Dutch to English by MediLingua Translations and is listed in Multimedia Appendix 1. The smartphone app MEASuRE (Maastricht Electronic Abdominal Symptom REporting), which in its current form was specifically developed for the use of the ESM in patients with endometriosis [13], was downloaded on participants’ smartphones and activated for the 7-day course of the study period. Subjects were instructed to carry their smartphone with them during this week. The MEASuRE app was set to send out an auditory and written signal 10 times a day at random moments between 07:30 AM and 10:30 PM. The ESM questionnaire, also called “beep assessment” or “beep questionnaire,” was available for 10 minutes after a signal, and after this time frame, questionnaires were considered as missing data if not completed. Therefore, subjects were instructed to complete as many questionnaires as possible each day, but to skip the questionnaire if answering the questions was not appropriate, for example, when driving a car.

#### End-of-Day and End-of-Week Symptom Questionnaires

The Brief Pain Inventory (BPI Short Version—Dutch language) was used as an end-of-day pain diary. An average pain score experienced over the last 24 hours was given on an 11-point NRS (0=*not at all* to 10=*very much so*) at the end of each study day (on paper) [26]. At the end of the 7-day study period, patients completed validated symptom questionnaires using an eCRF system (Castor EDC) [25]. The questionnaires included are listed in Table 1.

**Table 1:** Self-reported questionnaires for retrospective symptom assessment, used for validation of the endometriosis-specific ESM^a^ questionnaire.

Self-reported questionnaire	Content	Questions, n
Pain on NRS^b^	Abdominal pain, dysmenorrhea, and dyspareunia	3
EHP-30^c^ [12]	Health-related quality of life for patients with endometriosis. Items assess symptoms on 5 domains: pain, control and powerlessness, social support, emotional well-being, and self-image	30
GSRS-IBS^d^ [27]	Gastrointestinal symptoms. Items assess symptoms of pain, bloating, constipation, diarrhea, and satiety	13
PHQ-9^e^ [28]	Screening for subclinical depressive symptoms	9
GAD-7^f^ [29]	Screening for generalized anxiety disorder and assessment of severity	7

^a^ESM: experience sampling method.

^b^NRS: Numeric Rating Scale.

^c^EHP-30: 30-item Endometriosis Health Profile.

^d^GSRS-IBS: Gastrointestinal Symptom Rating Scale–Irritable Bowel Syndrome.

^e^PHQ-9: 9-question Patient Health Questionnaire.

^f^GAD-7: 7-item Generalized Anxiety Disorders Scale.

### Statistical Analysis

As the identical ESM questionnaire is repeatedly answered several times a day, we expected each patient to complete on average 5 of 10 assessments [13]. Power calculation resulted in a minimum of 25 patients who needed to complete the study protocol to validate this tool [30]. All analyses were performed using SPSS (SPSS Statistics for Windows, Version 25.0, Released 2017; IBM Corp.).

The compliance was calculated as the percentage of ESM questionnaires that were completed by all participants during the study period, per day, and measurement moment. Concurrent validity was assessed by comparing ESM scores with end-of-day pain scores (ie, on day level; no repeated measures within the day) and with end-of-week pain scores (ie, on week level; no repeated measures within the week). To compare ESM scores with end-of-day pain scores, the mean and maximum scores for the ESM were calculated for each of the 7 days, that is, combining all repeated measurements of each subject for the concerning day. Associations between end-of-day scores and ESM scores were tested using linear mixed effects models with the end-of-day score as the dependent and the mean ESM score as the independent variable, a random intercept, corrected for repeated measures using a first-order autoregressive (AR1) correlation structure. The level of agreement between end-of-day and ESM scores was evaluated by calculating intraclass correlation coefficients (ICCs), based on a single-rating consistency 2-way model. The ICCs that were calculated in this study were used to measure agreement between different scores that aimed to measure the same construct, such as end-of-week scores compared with mean ESM scores. To compare ESM scores with end-of-week questionnaire scores, all ESM measures were averaged to 1 score per subject. Differences between the measurement methods were tested using paired sample *t* tests, and Pearson correlation coefficients (*r*) were calculated. A value of *r* greater than 0.7 is considered a strong correlation, between 0.4 and 0.7 a moderate correlation, and anything less than 0.4 is considered a weak or no correlation [31]. For this matter, however, we notice that the paired *t* test cannot cope with the multilevel structure of the ESM pain scores. Nevertheless, to the best our knowledge, there is no alternative analysis that could cope with this while also comparing the multiple ESM scores with a day/week mean score. Results are displayed for creating insight; no conclusions will be made from significance testing.

The internal consistency of the ESM-PROM was evaluated by dividing the items into 4 symptom domains and calculating the Cronbach coefficient per domain.

## Results

### Study Population

In total, 64 women were recruited. Thirty-six patients were excluded as they did not meet inclusion criteria (n=6), declined participation (n=9), did not respond to e-mail or phone calls after providing the patient information leaflet (n=17), or had to quit the study because of technical phone problems (n=4). Of 28 patients who completed the study, 15 were recruited at the outpatient clinic and 13 were recruited by advertisement. Baseline characteristics are listed in Table 2.

**Table 2:** Baseline characteristics (N=28).

Characteristics		Values
Recruited by MUMC+,^a^ n (%)		15 (54)
Recruited by advertisement, n (%)		13 (46)
Age, mean (SD)		35.46 (9.39)
BMI, mean (SD)		25.97 (5.09)
**Educational level, n (%)**		
	High school	2 (7)
	College or university	26 (93)
**Work status, n (%)**		
	Student	2 (7)
	Unemployed	7 (25)
	Employed	19 (68)
**Relationship status, n (%)**		
	Single	7 (25)
	In relationship	21 (75)
**Parity, n (%)**		
	0	13 (46)
	≥1	15 (54)
Having a menstrual cycle, n (%)		17 (61)
**Use of hormones, n (%)**		22 (79)
	Oral contraceptives	5 (18)
	Levonorgestrel-releasing intrauterine system (Mirena)	9 (32)
	Progestins	8 (29)
	GnRH^b^	1 (4)
Regular use of pain medication, n (%)		20 (71)
Smoking, n (%)		6 (21)
Alcohol, n (%)		21 (75)
History of abdominal surgery for endometriosis, n (%)		20 (71)
Fertility treatment, n (%)		7 (25)
Current child wish, n (%)		6 (21)
Unplanned childlessness, n (%)		4 (14)
Traumatic life event, n (%)		3 (11)

^a^MUMC+: Maastricht University Medical Center+.

^b^GnRH: gonadotropin-releasing hormone.

### Compliance

Of all ESM morning questionnaires, 87.2% were completed and those took on average 18 seconds to complete. The completion rate of the ESM beep assessments was 52.1%, which corresponds to a mean number of completed assessments of 37 out of 70 per individual over the 7 days. It took participants on average 2 minutes 11 seconds to complete a beep questionnaire. The lowest number of completed measurements per subject was 19; the highest number was 63. Table 3 shows the mean percentage of completed ESM assessments per study day. The response rate was highest on the first 2 study days, with on average 65% of completed assessments. During the study period, the compliance decreased with the lowest number of completed assessments on the last study day (34%). Table 4 shows the mean percentage of completed ESM assessments per measurement moment during the day, with 1 indicating the first assessment of the day (morning) and 10 the last assessment of the day (evening). Response rates fluctuated between 39% and 56% during the day.

In total, 1.8% of questionnaires were started but not completed. The end-of-day pain questionnaire (on paper) was on average completed by 67.6%, and all participants completed the end-of-week questionnaire.

**Table 3:** Mean % completed ESMa assessments per study day.

Study days	Mean completion, %
1	64.6
2	65.4
3	59.6
4	56.1
5	45.7
6	38.9
7	34.3

^a^ESM: experience sampling method.

**Table 4:** Mean % of completed ESMa assessments per measurement moment during the day, averaged for all subjects and all 7 days during the study period.

Measurement moment	Mean completion, %
1	39.29
2	48.98
3	52.55
4	55.61
5	51.02
6	52.04
7	56.63
8	52.55
9	58.16
10	54.08

^a^ESM: experience sampling method.

### Concurrent Validity

#### End-of-Day Pain Score Compared With ESM Score

In this analysis, 19 patients were included, as 9 patients did not complete or return the end-of-day questionnaire. The average *mean* and *maximum* daily abdominal pain scores, as scored by the ESM, were 3.29 (SD 2.76) and 4.80 (SD 2.96), respectively. The average abdominal pain score in the end-of-day questionnaire was 4.31 (SD 2.32). Mean differences show a tendency to peak reporting as the *maximum* ESM scores show less difference to the end-of-day score than the *mean* ESM scores. When correcting for repeated measures (AR1 covariate structure), associations between end-of-day scores and corresponding *mean* and *maximum* ESM scores were significant. Furthermore, both ICCs between end-of-day diary scores and *mean* and *maximum* ESM scores demonstrate a good agreement between the 2 assessment methods, indicating that both measurement methods assess the same constructs (Table 5).

**Table 5:** Mean difference, association, and intraclass correlation coefficients (ICCs) between end-of-day diary scores and experience sampling method (ESM) mean and maximum scores for abdominal pain in 19 patients.^a^

	Mean difference			Association			ICCs	
	Difference	SE	Estimate		SE	ICC		95% CI
End-of-day vs ESM *mean* score	0.94	2.08	0.53^b^		0.08	0.80^b^		0.71-0.86
End-of-day vs ESM *maximum* score	–0.53	0.19	0.47 ^b^		0.06	0.81^b^		0.73–0.87

^a^Mean differences tested using the paired sample *t* test. A positive difference indicates a higher end-of-day score than the ESM. Significance of associations tested using mixed linear models with end-of-day diary scores as the dependent variable and ESM mean or maximum scores as the independent variable, corrected for repeated measures (first-order autoregressive covariate structure). The estimate indicates the strength and direction of the association.

^b^*P*<.001.

#### End-of-Week Scores Compared With ESM

The comparison and correlation between ESM and end-of-week pain scores scored on the NRS are shown in Table 6. End-of-week scores for abdominal pain and dysmenorrhea were significantly higher than mean ESM scores. However, the end-of-week score for abdominal pain did not differ significantly from ESM maximum scores, indicating peak level reporting at the end of the week. Dyspareunia scored by the ESM and at the end of the week were not significantly different. Pearson correlation showed strong and significant correlations for all pain scores (ie, abdominal pain, dysmenorrhea, and dyspareunia). The dysmenorrhea score for the ESM was calculated as the average abdominal pain score on days that patients experienced vaginal blood loss.

**Table 6:** End-of-week pain scores compared with experience sampling method (ESM) scores (mean scores on subject level).^a^

End-of-week vs ESM	End-of-week score, mean (SD)	ESM score, mean (SD)	Mean difference	SE	Pearson correlation coefficient (*r*)
Abdominal pain	4.96 (2.36)	3.21 (2.99); 4.71 (2.95)^b^	1.807; 0.371^b^	0.394; 0.524^b^	0.821; 0.830^b,c^
Dysmenorrhea	5.50 (2.59)	4.59 (3.18)	1.765	1.641	0.813^d^
Dyspareunia	2.69 (2.36)	2.63 (2.36)	–0.167	0.749	0.945^c^

^a^Differences were tested using the paired sample *t* test.

^b^Maximum (SD) values.

^c^*P*<.001.

^d^*P*<.05.

**Figure 1:**
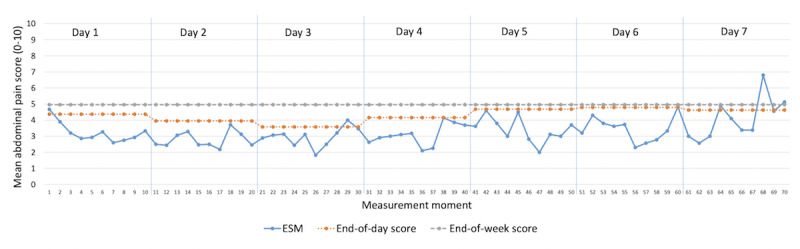
Abdominal pain scores (on an 11-point Numeric Rating scale) for ESM, end-of-day, and end-of-week scores over the 7-day study period, averaged for all participants. Each day, 10 assessments were available for the ESM; 1 assessment was completed at the end of each day and 1 assessment was completed at the end of the week. ESM: experience sampling method.

To visualize the comparison between the ESM and end-of-day as well as end-of-week scores in more detail, average abdominal pain scores for the ESM per measurement moment and the end-of-day and end-of-week scores are shown in Figure 1. This figure depicts a highly fluctuating pattern of abdominal pain during the 7-day study period when assessed using the ESM. The end-of-day scores are extrapolated to the entire day and the end-of-week scores to the entire week. The discrepancy between the assessment methods is highlighted here.

The correlation between ESM and end-of-week scores is shown in Table 7 and assessed by the EHP-30, Gastrointestinal Symptom Rating Scale–Irritable Bowel Syndrome (GSRS-IBS), 9-question Patient Health Questionnaire (PHQ-9), and 7-item Generalized Anxiety Disorders Scale (GAD-7), which do not allow the comparison between mean scores due to differences in scoring systems. Strong and significant correlations were found between the ESM and all symptoms scored by the GSRS-IBS, GAD-7, and PHQ-9, except for “feeling down,” which showed a significant but moderate correlation. Strong and significant correlations between the ESM and EHP-30 were found in 11 of 18 possible compared items (61%). Four items (22%) showed a moderately significant correlation, and for 3 items (17%), a small, nonsignificant correlation was found.

**Table 7:** Correlation between end-of-week scores and experience sampling method (ESM) scores (concurrent validity).

Symptoms		Pearson correlation coefficient (*r*)
**Correlation between EHP-30^a^ and ESM**		
	EHP-30: Been unable to go to social events because of the pain? ESM: My symptoms are getting in the way of my social activities	0.520^b^
	EHP-30: Been unable to do jobs around the home because of the pain? ESM: My symptoms are getting in the way of my household chores	0.222^c^
	EHP-30: Found it difficult to exercise or do the leisure activities you would like to do because of the pain? ESM: My symptoms are getting in the way of sports/hobbies	0.505^b^
	EHP-30: Found it difficult to stand because of the pain? ESM: I feel pain when I am standing	0.145^c^
	EHP-30: Found it difficult to sit because of the pain? ESM: I feel pain when I am sitting	0.554^b^
	EHP-30: Found it difficult to walk because of the pain? ESM: I feel pain when I am walking	0.446^d^
	EHP-30: Been unable to sleep properly because of the pain ESM: I slept well	0.606^b^
	EHP-30: Had to go to bed/lie down because of the pain? ESM: I feel that I need to rest	0.254^c^
	EHP-30: Been unable to do the things you want to do because of the pain? ESM: My symptoms are getting in the way of my activities	0.527^b^
	EHP-30: Generally felt unwell? ESM: I feel well physically	–0.502^b^
	EHP-30: Felt depressed? ESM: I feel dispirited (down)	0.729^e^
	EHP-30: Felt weepy/tearful? ESM: I feel emotional	0.748^b^
	EHP-30: Felt bad-tempered or short-tempered? ESM: I feel irritable	0.729^b^
	EHP-30: Couldn't do certain tasks at work because of the pain? ESM: My symptoms are getting in the way of work	0.466^d^
	EHP-30: Experienced pain during or after sexual intercourse? ESM: I suffered from pain during or after sexual intercourse	0.688^d^
	EHP-30: Has avoided intercourse because of the pain? ESM: I avoided sexual intercourse because of pain symptoms	0.466^d^
**Correlation between GSRS-IBS^f^ and ESM**		
	GSRS: Have you been bothered by nausea? ESM: I feel nauseous	0.803^e^
	GSRS: Has your stomach felt bloated? ESM: I feel discomfort due to being bloated	0.841^e^
	GSRS: Have you suffered from a visibly distended abdomen? ESM: My stomach is distended (swollen)	0.859^e^
	GSRS: Have you been bothered by abdominal pain? ESM: I suffer from abdominal pain	0.691^e^
**Correlation between PHQ-9/GAD-7^g^ and ESM**		
	PHQ-9: Feeling down, depressed, or hopeless ESM: I feel dispirited (down)	0.380^d^
	PHQ-9: Feeling tired or having little energy ESM: I feel… (very tired – full of energy)	–0.778^e^
	GAD-7: feeling nervous, anxious, or on edge ESM: I feel stressed	0.726^e^
	GAD-7: Worrying too much about different things ESM: I am worried	0.738^e^
	GAD-7: trouble relaxing ESM: I feel relaxed	–0.496^b^
	GAD-7: Becoming easily annoyed or irritable ESM: I feel irritable	0.533^b^

^a^EHP-30: 30-item Endometriosis Health Profile. The questionnaire starts with “During the last 4 weeks, how often, because of your endometriosis, have you….” Scale = 5-point Likert (Never – Always).

^b^*P*<.01 when differences were assessed using a paired samples *t* test.

^c^Not significant.

^d^*P*<.05 when differences were assessed using a paired samples *t* test.

^e^*P*<.001 when differences were assessed using a paired samples *t* test.

fGSRS-IBS: Gastrointestinal Symptom Rating Scale–Irritable Bowel Syndrome. Questions are asked over the past week. The scale used was a 7-point Likert scale (from no discomfort at all to Very severe discomfort).

gPHQ-9: 9-question Patient Health Questionnaire; GAD-7: 7-item Generalized Anxiety Disorder Scale. The questionnaires start with “Over the last 2 weeks, how often have you been bothered by any of the following problems?” The scale is a 4-point Likert scale (from Not at all to Nearly every day).

### Internal Consistency

To determine the internal consistency of the ESM-PROM for endometriosis, items were categorized into 4 constructs: abdominal symptoms, general somatic symptoms, positive affect, and negative affect. Cronbach coefficients for each of these domains are shown in Textbox 1. Results demonstrate a good internal consistency for abdominal symptoms, general somatic symptoms, and positive affect, and an excellent internal consistency for negative affect.

**Internal consistency for 4 symptom domains within the experience sampling method patient-reported outcome measure, reflected by the Cronbach coefficient.**

**Abdominal symptoms (Cronbach =.793)**

Abdominal pain, bloating, distended abdomen, urge to defecate, vaginal blood loss

**General somatic symptoms (Cronbach =.767)**

Dizziness, nausea, headache, muscle pain, shortness of breath

**Mental—positive affect (Cronbach =.716)**

Cheerful, relaxed

**Mental—negative affect (Cronbach =.935)**

Dispirited/down, emotional, stressed, worried, irritable

## Discussion

### Principal Findings

This study evaluated the concurrent validity and internal consistency of a previously developed PROM based on the ESM principle for momentary symptom assessment in endometriosis [13]. In this study, we demonstrated that ESM abdominal pain scores in women with endometriosis are strongly correlated with the corresponding end-of-day and end-of-week pain scores. However, end-of-week scores were significantly higher than *mean* ESM scores but did not differ significantly from the *maximum* ESM level during the day. This indicates peak reporting of pain scores after a period of recall. Although we notice that the paired *t* test cannot cope with the multilevel structure of the ESM pain scores and results should be interpreted with caution, the phenomenon of peak reporting was also described earlier in an irritable bowel syndrome population [16].

ESM scores showed strong concurrent validity when compared with symptoms scored by the GSRS-IBS, GAD-7, and PHQ-9, except for a significant moderate correlation for the symptom “feeling down.” This is probably due to the use of different words to express a depressed mood by the ESM (“feeling dispirited, down”) versus PHQ (“feeling down, depressed, hopeless”). Furthermore, the present mental state of a person filling in a retrospective questionnaire might impact the result (psychological bias). The correlation for “feeling down” as assessed by ESM and EHP-30 was strong. Concerning the comparison of the ESM with EHP-30, social and behavioral items were less strongly correlated than the occurrence and severity of physical and psychological symptoms. For example, nonsignificant correlations between the ESM and EHP-30 were found for the experience of being unable to do household chores, difficulty standing, and the need to rest because of symptoms. This could be explained by the fact that social or behavioral factors are less suitable for psychometric evaluation as they are not scored on a fixed scale but either occur or do not. In addition, behavioral and social items might be impacted more strongly by ecological and recall biases. This strengthens the advantages of the ESM tool, as social and behavioral responses to symptoms are important to estimate disease burden. Cronbach coefficients demonstrated a good internal consistency for abdominal symptoms, general somatic symptoms and positive affect, and an excellent internal consistency for negative affect within the ESM-PROM. As the ESM is meant to measure fluctuations within symptoms and experiences, a test-retest reliability is not a good measure of reproducibility of ESM data. Therefore, no test-retest reliability analyses were performed.

A strength of this study is that the evaluated tool was previously designed based on specific recommendations for PROM development [32]. Only 1.8% of questionnaires were started but not completed, indicating that the questionnaire was not too time-consuming. A large number of repeated measures significantly increase the power of the analyses, and the criteria for power calculation, as advised by Schuster et al [30], were met. As the ESM questionnaire is made available by a smartphone app, a potential limitation is that the compliance depends on technical issues, such as signal blocking and network failure by the data company. However, the app does not need an internet connection as it functions offline. Smartphone holders using a specific type of smartphone experienced blocking of ESM messages that could not be addressed, and thus, they could not complete the study (n=4). Another drawback of the ESM is the patient burden, as the repeated assessments during the day are more time-consuming than most conventional assessment methods. This may result in low compliance.

However, in this study, 52% of the total assessments were completed, which is considerably higher than the generally accepted completion rates of 33% in other studies [33,34]. Although 2 of 28 individual patients with endometriosis completed only 19 measurements out of 70 (=27.1% and therefore did not meet the 33% criterion), they were not excluded from the study. Furthermore, a variable number of questions of the ESM assessment due to trigger questions could result in giving negative answers to prevent follow-up questions and therefore win time. As opposed to frequently used static measures, which describe health status at a specific time point, the ESM provides dynamic assessments of symptomatology, allowing for the detection of symptom fluctuation and symptom formation, that is, how symptoms impact symptoms. Given that endometriosis is a heterogeneous disorder often presenting with psychological disturbances or general somatic complaints, which is also reflected by lower levels of quality of life [9], items were included on mental state and frequently reported physical complaints in addition to endometriosis-specific symptoms. Furthermore, environmental and behavioral items were added as they represent and influence disease burden. The assessment of patients’ mental states using the ESM has been thoroughly described in psychiatric and psychosomatic research, also concerning transdiagnostic issues [14,17-24,35,36]. Accordingly, the ESM yields detailed data of the dynamics of endometriosis symptoms in daily life, which could be used to support self-insight, shared decision-making, and prediction of treatment satisfaction in clinical practice. This may also lead to individualized treatment strategies that can improve quality of life and decrease costs [14,17]. The present endometriosis-specific ESM questionnaire was developed in Dutch and was officially translated by following the translation guidelines [37]. Official English language validation is currently planned.

### Conclusions

This study shows the validity and reliability of the newly developed electronic instrument for the momentary measurement of symptoms in women with endometriosis, based on the ESM. Compliance for answering the ESM questions was as high as 52%. We demonstrated peak reporting of pain scores after a period of recall since end-of-week pain scores were significantly higher than *mean* ESM scores but did not differ significantly from the *maximum* ESM level during the day. This ESM-PROM has the advantage of providing a more detailed view on individual symptom patterns, with the option to analyze interactions between symptoms, the psychological state, and environmental factors, to aid disease monitoring in both clinical practice and research settings. The ESM could offer the possibility of self-insight of patients in their symptomatology, leading to more individualized treatment strategies and shared decision-making in treatment choices, which can considerably improve the quality of life of women suffering from endometriosis.

## Appendix

Multimedia Appendix 1 Table S1. Set of questions (q) for the endometriosis specific ESM-PROM [13].

## Abbreviations

AR1: first-order autoregressive
CPP: chronic pelvic pain
eCRF: electronic clinical case report form
EDC: electronic data capture
EHP-30: 30-item Endometriosis Health Profile
ESM: experience sampling method
GAD-7: 7-item Generalized Anxiety Disorders Scale
GSRS-IBS: Gastrointestinal Symptom Rating Scale–Irritable Bowel Syndrome
ICC: intraclass correlation coefficient
MEASuRE: Maastricht Electronic Abdominal Symptom REporting
MUMC+: Maastricht University Medical Center+
NRS: Numeric Rating Scale
PHQ-9: 9-question Patient Health Questionnaire
PROM: patient-reported outcome measure
