# Supplementary material for: Real-time Symptom Assessment in Patients With Endometriosis: Psychometric Evaluation of an Electronic Patient-Reported Outcome Measure, Based on the Experience Sampling Method
Source: JMIR Form Res. 2023 Mar 3;7:e29480. doi: 10.2196/29480 (PMC10024211; doi:10.2196/29480)
Supplement: Multimedia Appendix 1 [file formative_v7i1e29480_app1.pdf]

Multimedia Appendix

Table 1. *Set of questions (q) for the endometriosis specific ESM-PROM [13]*

One-off questionnaire	Answer options
**1. The following applies to me:**	I have regular periods, My periods are irregular, I never experience blood loss<br>** in case of regular or irregular periods, q1.1 is asked.*<br>*** in case of never experiencing blood loss, q3 (endometriosis specific symptoms) is not asked.*
**1.1 How many days ago did your most recent period start?**	0 t/m 31 days.
**Morning questionnaire**	
**Sleep**	**Answer options**
**1. I slept well**	0 (not at all)—10 (very much so)
**2. I had trouble falling asleep.**	Yes – No
**3. I woke up during the night.**	Yes – No<br>** in case of "Yes", q3.1 is asked.*
**3.1 I woke up because of…**	An urge to urinate, abdominal pain, worrying, something else/no apparent cause.
**Sexuality**	**Answer options**
**4. I had sexual intercourse.**	Yes – No<br>** in case of "Yes", q4.2 is asked.*<br>*** in case of "No", q4.1 is asked.*
**4.1 I avoided sexual intercourse because of pain symptoms.**	Yes – No
**4.2 I suffered from pain during or after sexual intercourse.**	0 (no pain)—10 (a lot of pain)<br>** in case of "1 or more", q4.3 is asked.*
**4.3 The pain was …**	Superficial vaginal pain, deep vaginal pain, abdominal pain<br>*~More than one answer option possible.*
**Momentary assessment**	
**Endometriosis specific symptoms**	**Answer options**
**1. I suffer from abdominal/pelvic pain**	0 (no pain)—10 (a lot of pain)<br>** in case of "1 or more", q1.1 is asked.*

**1.1 I feel this pain in the following place(s) in my abdomen:**	0 – 9<br>**R** **L**<br>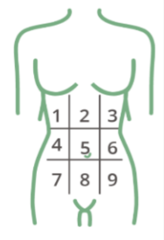
**2. I suffer from pain in my...**	Shoulders, groins, back, anus, legs, other area, none of the above.<br>*~More than one answer option possible.*
**2.1 I feel pain when I am…**	Walking, sitting, standing, laying down.<br>*~More than one answer option possible*
**3. The vaginal blood loss is as follows:**	No bleeding, spotting, light bleeding, moderate bleeding, heavy bleeding.
**4. I feel…**	Cold (-5) – neutral (0) – very hot/hot flushes(+5)
**5. Since the last questionnaire I have urinated...**	0 times, once, twice, 3 times, 4 times, > 4 times.<br>** In case of "once or more", q5.1 is asked.*
**5.1 When urinating I experienced the following symptoms:**	Pain while passing urine, strong urge to urinate, blood loss while passing urine, difficulty passing urine, no symptoms.<br>** In case of "strong urge to urinate", q5.2 is asked.*<br>*~More than one answer option possible*
**5.2 How strong was the urge to urinate.**	I could put it off for 30 minutes, I could put it off for 10 minutes, I had to urinate instantly/I could not put it off any longer.
**6. I feel discomfort due to being bloated**	0(not at all) – 10(very much so)
**7. My stomach is distended (swollen).**	0(not at all) – 10(very much so)
**8. I feel an urge to defecate.**	0(not at all) – 10(very much so)
**9. Since the last questionnaire I have had… bowel movements.**	0, 1, 2, 3, 4, 5, >5<br>**In case of "1 or more", q9.1 is asked.*
**9.1 The appearance of my stools was as follows:**	**Bristol Stool Form Scale**<br>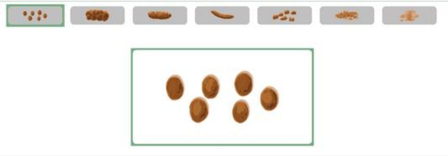
**9.2 My bowel movements are painful**	0(not at all) – 10(very much so)

**9.3 I have rectal bleeding/blood loss.**	0 (not at all)—10 (very much so)
**General somatic symptoms**	**Answer options**
**10. I feel dizzy.**	0 (not at all)—10 (very much so)
**11. I feel nauseous.**	0 (not at all)—10 (very much so)
**12. I have a headache.**	0 (not at all)—10 (very much so)
**13. I feel short of breath.**	0 (not at all)—10 (very much so)
**14. I have sore muscles and/or joints.**	0 (not at all)—10 (very much so)
**15. I feel…**	-5 (very tired) – 0 (neutral) – +5 (full of energy)
**16. I feel well physically.**	0 (not at all)—10 (very much so)
**Mood and psychological factors**	**Answer options**
**17. I feel happy.**	0 (not at all)—10 (very much so)
**18. I feel sad (down).**	0 (not at all)—10 (very much so)
**19. I feel emotional.**	0 (not at all)—10 (very much so)
**20. I feel stressed.**	0 (not at all)—10 (very much so)
**21. I feel relaxed.**	0 (not at all)—10 (very much so)
**22. I am worried.**	0 (not at all)—10 (very much so)
**23. I feel irritable.**	0 (not at all)—10 (very much so)
**Social and contextual factors**	**Answer options**
**24. My current situation is as follows:**	I am at home, I am at someone else's home, I am at work/school, I am in a public place, I am on the move, I am somewhere else.
**25. Just before the questionnaire I was…**	Resting, at work/school, doing household chores/grocery shopping, taking care of children/providing other informal care, eating/drinking, relaxing/spending time on a hobby, exercising/participating in sports, on the move, doing something else.
**26. I feel uneasy/easy in this situation.**	-5 (very uneasy) – 5 (very easy)
**27. My symptoms are getting in the way of my activities.**	0 (not at all)—10 (very much so)<br>** In case of "1 or more", q27.1 is asked.*
**27.1 My symptoms are negatively affecting my…**	Household chores, sports/hobbies, work, social activities.<br>*~More than one answer option possible.*

**28. I am currently in the company of…**	My partner, children, friends, housemates, co-workers, relatives (who are not part of my household), acquaintances, people I don’t know/others, a big group, one or more pets, no one.<br>** In case “no one” is NOT answered, q28.1 is asked.*
**28.1 I feel uneasy/easy in this company.**	-5 (very uneasy) – 5 (very easy)
**29. I feel that I need to rest.**	0 (not at all)—10 (very much so)
**Use of nutrition and medication**	**Answer options**
**30. Since the last questionnaire I have eaten…**	Breakfast, lunch, dinner, a snack/fruit, nothing
**31. Since the last questionnaire I have taken/had…**	Caffeine (coffee), nicotine (smoking), alcohol, recreational drugs, painkillers, none of the above.<br>** In case of “alcohol”, q31.1 is asked.*<br>*** In case of “painkillers”, q31.2 and q31.3 are asked.*
**31.1 Since the last questionnaire I have had … units of alcohol.**	0-2, 3-4, > 4
**31.2 Since the last questionnaire I have used painkillers for…**	Abdominal pain and/or pelvic pain, something else.
**31.3 Since the last questionnaire I have used the following painkiller ...**	Paracetamol, an NSAID (ibuprofen, Nurofen, aspirin, mefenamic acid), tramadol/Zaldiar, an opioid (e.g. morphine), homoeopathic preparations, CBD oil (cannabis oil)/ hashish/ THC.
